# Supplementary material for: Cost-analysis of COVID-19 sample collection, diagnosis, and contact tracing in low resource setting: The case of Addis Ababa, Ethiopia
Source: PLoS One. 2022 Jun 9;17(6):e0269458. doi: 10.1371/journal.pone.0269458 (PMC9182302; doi:10.1371/journal.pone.0269458)
Supplement: S1 Appendix — (DOCX) [file pone.0269458.s001.docx]

**S1 Appendix**

**I. Inputs and data sources**

**S1 Table: Source of data**

| *Data type* | *Source of information* |
| --- | --- |
| Salary | EPHI, finance directorate, Laboratory unit (parasitology department) |
| Equipment | EPHI laboratory unit, contact tracing unit, sample collectors |
| Food cost | EPHI laboratory unit and contact tracing unit |
| Vehicle | EPHI transport unit and laboratory unit, |
| Supplies | Laboratory unit, contact tracing unit, sample collectors |
| Rental price | Average rental value per meter square at EPHI neighborhood |

**S2 Table: Laboratory supplies used for COVID-19 diagnosis, June-December 2020**

| *Item type* | *Contributor* | *Unit of measurement* | *Amount used* |
| --- | --- | --- | --- |
| Gloves | EPHI | box | 34 |
| N-95 mask | EPHI | pcs | 60 |
| Gown (disposable apron) | EPHI | pcs | 330 |
| Permanent Marker | EPHI | pcs | 4 |
| 70% Ethanol | EPHI | Liter | 4 |
| 1% Bleach | EPHI | Liter | 30 |
| Face mask | EPHI | pcs | 240 |
| Pippete tip (200µl & 1000µl) | EPHI | rack | 1500 |
| Micro Centrifuge tip | EPHI | pack | 3 |
| Ethanol solution for extraction | EPHI | bottle | 4 |
| 8-tube strips | EPHI | pcs | 150 |
| Extraction kit-May | EPHI | Box | 4,962 4,962 |
| Extraction kit-June | EPHI | Box | 10,541 10,541 |
| Extraction kit-July | EPHI | Box | 9,689 9,689 |
| Extraction kit-August | EPHI | Box | 13,071 13,071 |
| Extraction kit-September | EPHI | Box | 6,075 6,075 |
| Extraction kit-October | EPHI | Box | 9,443 9,443 |
| Extraction kit-November | EPHI | Box | 10,834 9,340 |
| Extraction kit-December | EPHI | Box | 4,962 10,834 |
| Real-time fluorescent rt-pcr kit for detecting sars-cov-2_May | EPHI | Box | 10,541 10,541 |
| Real-time fluorescent rt-pcr kit for detecting sars-cov-2_June | EPHI | Box | 9,689 9,689 |
| Real-time fluorescent rt-pcr kit for detecting sars-cov-2_July | EPHI | Box | 13,071 13,071 |
| Real-time fluorescent rt-pcr kit for detecting sars-cov-2_August | EPHI | Box | 6,075 6,075 |
| Real-time fluorescent rt-pcr kit for detecting sars-cov-2_Septmeber | EPHI | Box | 9,443 9,443 |
| Real-time fluorescent rt-pcr kit for detecting sars-cov-2_October | EPHI | Box | 10,834 9,340 |
| Real-time fluorescent rt-pcr kit for detecting sars-cov-2_November | EPHI | Box | 9,340 9,340 9,44 |
| Real-time fluorescent rt-pcr kit for detecting sars-cov-2_December | EPHI | Box | 10,834 10,834 |

**S3 Table: Equipment used for laboratory diagnosis, June-December 2020**

| *Equipment (list)* | *Contributor* | *Quantity* | *Useful period* |
| --- | --- | --- | --- |
| Vortex mixers | EPHI | 3 | 5 |
| Biosafety cabinet (the baker company) | EPHI | 1 | 5 |
| Pippete (1000) | EPHI | 4 | 3 |
| Pippete (500) | EPHI | 2 | 3 |
| Pippete (50-200) | EPHI | 4 | 3 |
| Hot plate for lysis | EPHI | 2 | 5 |
| Centrifuge 5424 R | EPHI | 2 | 5 |
| Donning shelf | EPHI | 1 | 5 |
| Refrigerator (2-8oC) | EPHI | 2 | 5 |
| Stabilizer | EPHI | 1 | 3 |
| Qantstudio5 (5 probes) | EPHI | 1 | 5 |
| AB7500real time PCR system | EPHI | 1 | 5 |
| Bench top laminal flow | EPHI | 2 | 5 |
| Printer | EPHI | 1 | 5 |
| Computer | EPHI | 3 | 5 |

**S4 Table: Building space used for COVID-19 testing, June-December 2020**

| *Building* | *Contributors* | *Surface area (m^2^)* | *Rental market price equivalent per m^2^ per year* | *Total number of days used for COVID-19* |
| --- | --- | --- | --- | --- |
|  |  |  |  |  |
| Parasitology (COVID-19) lab | EPHI | 100 | 3600 | 30 |
| Laboratory head | EPHI | 25 | 3600 | 30 |

**S5 Table: Vehicle used for laboratory personnel transportation, June-December 2020**

| *Vehicles* | *Contributors* | *Rent per day (ETB)* | *Number of days used per month* | *hours used* | *Number of car per day* | *Fuel cost (ETB)* |
| --- | --- | --- | --- | --- | --- | --- |
| HZJ 76 | Rental | 1913 | 30 | 6 | 1 | 10572.9 |

**S6 Table: Food cost for laboratory personnel (only dinner starting from September), June-December 2020**

| *Time of the day* | *Food cost per serving (ETB)* | *Number servings per day* | *Number of days per month* | *Total cost (ETB)* |
| --- | --- | --- | --- | --- |
| Breakfast | 400 | 15 | 30 | 180,000 |
| Dinner | 400 | 10 | 30 | 120,000 |

**S7 Table: Supplies used for contact tracing activities, June-December 2020**

| ***Item*** | ***Contributors*** | ***Unit of measurement*** | ***Quantity consumed*** |
| --- | --- | --- | --- |
|  |  |  |  |
| N-95 |  | pcs | 150 |
| Face mask |  | Pcs | 15660 |
| sanitizer (hand rub) |  | 100 ml | 150 |
| Total cost |  |  |  |

**S8 Table: Equipment used for contact tracing activities, June-December, 2020**

| **Equipment (list)** | **Contributor** | **Quantity** | **Purchase price** | **Useful period** | **No. days used for COVID-19** |
| --- | --- | --- | --- | --- | --- |
| **Tablets** | CSA | 54 | 9000 | 5 | 213 |

**S9 Table: Building used for contact tracing activities, June-December 2020**

| **Building size, square meter** | **Contributors** | **Rental value per square meter** | **number of months** | **% allocation to contact tracing** |
| --- | --- | --- | --- | --- |
|  |  |  |  |  |
| 100 | EPHI | 300 | 1 | 0.1 |

**S10 Table: Vehicle used for contact tracing activities, June-December, 2020**

| **Vehicles** | **Contributors** | **rent per day** | **number of days used** | **Number of car per day** | **Fuel cost (birr)** |
| --- | --- | --- | --- | --- | --- |
|  |  |  |  |  |  |
|  |  |  |  |  |  |
| HZJ 76 | EPHI/rental | 1967 | 30 | 26 | 10572.92308 |
| HZJ 78 | EPHI/rental | 1928 | 30 | 10 | 10572.92308 |
| HZJ 105 | EPHI/rental | 1544 | 30 | 10 | 10572.92308 |
| HZJ 76 | EPHI/rental | 1967 | 30 | 1 | 10572.92308 |
| HZJ 78 | EPHI/rental | 1928 | 30 | 1 | 10572.92308 |
| HZJ 105 | EPHI/rental | 1544 | 30 | 1 | 10572.92308 |

**S11 Table: Supplies used for sample collection activities, June-December 2020**

| **Item** | **Contributors** | **Unit of measurement** | **Quantity consumed** |
| --- | --- | --- | --- |
|  |  |  |  |
| Surgical gloves | EPHI | Box of 100 | 16.2 |
| disposable gloves | EPHI | Box of 100 | 16.2 |
| Gown (disposable apron) | EPHI | PCS | 540 |
| N-95 mask | EPHI | PCS | 540 |
| Hand sanitizer-100 ml | EPHI | Bottle | 8 |
| Face shield goggles: plastic | EPHI | box of 50 | 10.8 |
| Sample collection tube-June | EPHI | Tube | 3335 |
| Sample collection tube-July | EPHI | Tube | 8152 |
| Sample collection tube-August | EPHI | Tube | 18609 |
| Sample collection tube-September | EPHI | Tube | 9527 |
| Sample collection tube-October | EPHI | Tube | 8565 |
| Sample collection tube-November | EPHI | Tube | 9447 |
| Sample collection tube-December | EPHI | Tube | 10943 |

**S12 Table: Vehicle used for sample collection activities, June-December 2020**

| **Vehicles** | **Contributors** | **rent per day** | **number of days used** | **Number of car per day** | **Fuel cost (birr)** |
| --- | --- | --- | --- | --- | --- |
|  |  |  |  |  |  |
| HZJ 76 | EPHI/rental | 1967 | 30 | 14 | 10572.92308 |
| HZJ 78 | EPHI/rental | 1928 | 30 | 8 | 10572.92308 |
| HZJ 105 | EPHI/rental | 1544 | 30 | 5 | 10572.92308 |

**S13 Table: Building used for sample collection activities, June-December 2020**

| **Building size, square meter** | **Contributors** | **Rental value per square meter** | **number of months** | **% allocation to contact tracing** |
| --- | --- | --- | --- | --- |
|  |  |  |  |  |
| 100 | EPHI | 300 | 1 | 0.1 |

**II. Additional results tables**

**S14 Table: COVID -19 sample collection costs in USD, June-December 2020**

| *Month* | *Number of samples collected* | *Number of COVID-19 positive samples* | *Personnel cost* | *Supplies cost* | *Building cost* | *Vehicle* | *Total cost* |
| --- | --- | --- | --- | --- | --- | --- | --- |
| June | 14,763 | 3,335 | 61,188 | 3,210.16 | 4.10 | 43,662 | 108,064 |
| July | 72,394 | 8,152 | 61,188 | 6,322.24 | 4.10 | 43,662 | 111,176 |
| August | 121,691 | 18,609 | 61,188 | 8,984.27 | 4.10 | 43,662 | 113,838 |
| September | 93,929 | 9,527 | 61,188 | 7,485.13 | 4.10 | 43,662 | 112,339 |
| October | 88,229 | 8,565 | 75,398 | 7,177.33 | 4.10 | 43,662 | 126,241 |
| November | 94,760 | 9,447 | 61,188 | 7,530.00 | 4.10 | 43,662 | 112,384 |
| December | 112,736 | 10,943 | 61,188 | 8,500.70 | 4.10 | 43,662 | 113,355 |
| ***Total*** | ***598,502*** | ***68,578*** | ***442,526*** | ***49,209.83*** | ***28.72*** | ***305,633*** | ***797,398*** |

**S15 Table: Number of COVID-19 contacts traced and cost of contact tracing in USD, June-December 2020**

| *Month* | *Number of contact traced* | *Number of positives identified* | *Personnel cost* | *Supply cost* | *Equipment cost* | *Building cost* | *Vehicle cost* | *Total cost* |
| --- | --- | --- | --- | --- | --- | --- | --- | --- |
| June | 69,559 | 2,992 | 83,865 | 4,376 | 147 | 5.27 | 440,098 | 528,490 |
| July | 144,923 | 8,495 | 129,158 | 4,376 | 147 | 5.27 | 440,098 | 573,783 |
| August | 151,225 | 9,166 | 129,158 | 4,376 | 147 | 5.27 | 440,098 | 573,783 |
| September | 158,332 | 12,281 | 129,158 | 4,376 | 147 | 5.27 | 440,098 | 573,783 |
| October | 179,540 | 14,809 | 25,058 | 4,376 | 147 | 5.27 | 10,964 | 40,550 |
| November | 182,270 | 16,176 | 25,058 | 4,376 | 147 | 5.27 | 10,964 | 40,550 |
| December | 184,837 | 17,240 | 25,058 | 4,376 | 147 | 5.27 | 10,964 | 40,550 |
| ***Total*** | ***1,070,686*** | ***81,159*** | ***546,512*** | ***30,630*** | ***1,026*** | ***36.92*** | ***1,793,283*** | ***2,371,489*** |

**S16 Table: Number of laboratory tests and laboratory diagnosis costs in USD, June-December 2020**

| *Month* | *Number of Samples* | *Number of positive* | *Personnel cost* | *Supplies cost* | *Equipment cost* | *Building cost* | *Food cost* | *Vehicles cost* | *Total cost* |
| --- | --- | --- | --- | --- | --- | --- | --- | --- | --- |
| May | 4,962 | 69 | 23,593 | 3,728 | 1,136 | 1,055 | 8,439 | 1,434 | 39,385 |
| June | 10,541 | 859 | 23,593 | 5,960 | 1,136 | 1,055 | 8,439 | 1,434 | 41,616 |
| July | 9,689 | 870 | 23,593 | 5,619 | 1,136 | 1,055 | 8,439 | 1,434 | 41,275 |
| August | 13,071 | 1,388 | 23,593 | 6,972 | 1,136 | 1,055 | 8,439 | 1,434 | 42,628 |
| September | 6,075 | 1,645 | 23,593 | 4,173 | 1,136 | 1,055 | 3,376 | 717 | 34,050 |
| October | 9,443 | 1,009 | 18,066 | 5,520 | 1,136 | 1,055 | 3,376 | 717 | 29,869 |
| November | 9,340 | 941 | 18,066 | 5,479 | 1,136 | 1,055 | 3,376 | 717 | 29,828 |
| December | 10,834 | 887 | 18,066 | 6,077 | 1,136 | 1,055 | 3,376 | 717 | 30,426 |
| Total | 73,955 | 7,668 | 172,164 | 43,528 | 9,087 | 8,439 | 47,257 | 8,603 | 289,078 |
