# Supplementary material for: Cost-analysis of COVID-19 sample collection, diagnosis, and contact tracing in low resource setting: The case of Addis Ababa, Ethiopia
Source: PLoS One. 2022 Jun 9;17(6):e0269458. doi: 10.1371/journal.pone.0269458 (PMC9182302; doi:10.1371/journal.pone.0269458)
Supplement: Attachment — Submitted filename: Response to reviewers.docx [file pone.0269458.s003.docx]

May 15, 2022

Dr. Sanjay Kumar Singh Patel
Academic Editor
PLOS ONE

Dear Editors,

We are submitting our revised manuscript entitled **"Cost-analysis of COVID-19 sample collection, diagnosis, and contact tracing in low resource setting: the case of Addis Ababa, Ethiopia " (PONE-D-21-20233)** to *PLOS ONE.*

We would like to thank the editor and reviewers for the constructive comments and suggestions which have helped to strengthen our work substantially.

Below, we have provided a detailed point-by-point response to all comments and suggestions raised in the review.

Thank you very much for your consideration of our manuscript.

Sincerely,

Amanuel Yigezu, on behalf of the authors

**Point-by-point response to the reviewers’ comments**

***Reviewer #1****:*

*The authors have addressed my previous comments adequately and I recommend publication with the following minor revisions. Recommendations for the authors are to present the cost results in the same order as they are mentioned in the methods and to make sure the abstract method and results are consistent with this as well. This will add clarity in reading the results.*

**Authors’ response: We thank the reviewer for his/her positive comments on our manuscript. We have now presented the cost results in the same order as they are mentioned in the methods both in the abstract and methods sections.**

*Secondly, the authors need to report all costs as they are shown on the figures. For example, personnel costs are separate from food costs in figure 2, so this should be the way it is reported in text.*

**Authors’ response: Thank you for the comment. We have now reported food costs in figure 2 the way it is reported in text.**

*Lastly, there are a few grammatical errors and misspellings throughout this version and I recommend the authors review for grammatical errors and edit as needed prior to publication*

**Authors’ response: Thank you for the comment. We have now edited the manuscript to correct grammatical error.**
